# Spontaneous Switching among Conformational Ensembles in Intrinsically Disordered Proteins

**DOI:** 10.3390/biom9030114

**Published:** 2019-03-22

**Authors:** Ucheor B. Choi, Hugo Sanabria, Tatyana Smirnova, Mark E. Bowen, Keith R. Weninger

**Affiliations:** 1 Department of Molecular and Cellular Physiology, Department of Neurology and Neurological Sciences, Department of Structural Biology, Department of Photon Science, Howard Hughes Medical Institute, Stanford University, Stanford, CA 94305, USA; ucheor@stanford.edu; 2 Department of Physics and Astronomy, Clemson University, Clemson, SC 29634, USA; hsanabr@clemson.edu; 3 Department of Chemistry, North Carolina State University, Raleigh, NC 27695, USA; tismirno@ncsu.edu; 4 Department of Physiology and Biophysics, Stony Brook University, Stony Brook, NY 11794, USA; 5 Department of Physics, North Carolina State University, Raleigh, NC 27695, USA

**Keywords:** dynamic configuration, free energy landscape, intrinsically disordered protein, IDP

## Abstract

The common conception of intrinsically disordered proteins (IDPs) is that they stochastically sample all possible configurations driven by thermal fluctuations. This is certainly true for many IDPs, which behave as swollen random coils that can be described using polymer models developed for homopolymers. However, the variability in interaction energy between different amino acid sequences provides the possibility that some configurations may be strongly preferred while others are forbidden. In compact globular IDPs, core hydration and packing density can vary between segments of the polypeptide chain leading to complex conformational dynamics. Here, we describe a growing number of proteins that appear intrinsically disordered by biochemical and bioinformatic characterization but switch between restricted regions of conformational space. In some cases, spontaneous switching between conformational ensembles was directly observed, but few methods can identify when an IDP is acting as a restricted chain. Such switching between disparate corners of conformational space could bias ligand binding and regulate the volume of IDPs acting as structural or entropic elements. Thus, mapping the accessible energy landscape and capturing dynamics across a wide range of timescales are essential to recognize when an IDP is acting as such a switch.

## 1. Introduction

In general, proteins do not fold into static structures. Rather, most proteins fluctuate within a pseudocontinuum of accessible configurations about their lowest energy folded state. Often, these fluctuations are coupled to the protein’s functional cycle such as catalytic activity or molecular recognition. However, many proteins lack a predominant low-energy state, and instead sample a broad and disparate ensemble of configurations. Such intrinsically disordered proteins (IDPs) lack the stable folded state, which is a central element in the classical structure–function paradigm. Nonetheless, IDPs, and proteins containing intrinsically disordered regions (IDRs), are now understood to play essential roles in cell signaling pathways and regulatory networks [1,2,3,4,5,6].

Models for how function arises from an ensemble of rapidly interconverting IDP conformers generally fall into two categories: those directly involved in molecular recognition and those acting as linker or structural elements. Unlike molecular recognition by folded domains, wherein the interaction residues are spread across the polypeptide chain, IDP interactions tend to involve short linear motifs (SLiMs), which are contiguous within the chain. Disordered SLiMs adopt an ensemble of conformations and these different conformations of the same sequence may be recognized by distinct binding partners [7]. The timescale of IDP conformational dynamics is an important determinant of the binding mode for IDPs. Rapid conformational dynamics supports the classic induced fit model, where the IDP can reconfigure the binding site before the initial encounter complex dissociates. Many IDPs couple the energy from order transitions to the recognition event, which provides a powerful mechanism for allostery. In other cases, the disorder persists even in the bound state [8,9]. If the timescale for IDP dynamics slows, then the binding mode is limited to conformational selection wherein the interaction is only possible during those windows when the binding competent configuration is present. In this way, the timescale of IDP conformational dynamics plays a key role in differentiating modes of molecular recognition by IDPs.

The dynamic nature of IDPs and IDRs makes them well suited to function as linkers between functional elements such as binding sites or folded domains. For example, the fly-casting model revealed how conformational exchange allows IDPs to explore a large volume in the search for binding partners [10,11,12]. The entropic clock model showed how the degree of extension of the IDP linker between a pore and its blocking domain controlled the open time of an ion channel [13]. The entropic bristle model suggested that IDPs can fill large volumes of three-dimensional (3D) space during their conformational searching, which can regulate protein interactions [14]. Thus, the timescale and the range of conformational sampling within the ensemble governs the structural properties of IDPs acting as linkers or bristles. 

These functional roles for IDPs rely on their dynamic properties to control the energetics of binding reactions as well as for regulating hydrodynamic volume and spacing. Understanding protein structure is the key to describing its function. With IDPs this necessitates extending our understanding beyond minimum energy states to further characterize the ensemble both in terms of the accessible landscape as well as the timescales of conformational dynamics. 

The rise of polymer science led to a great foundation of Nobel prize-winning theory to describe the behavior of homopolymers composed of many repeated subunits [15]. Based on different starting assumptions about the polymer, a continuum ensemble of end-to-end distances can be estimated by well-established approximations such as a Gaussian chain, a self-avoiding random coil, or a worm-like chain model [16,17]. While fully appropriate for polymers, in application to IDPs, such models lack molecular detail and rely on assumptions about polymer behavior that are not strictly applicable to polypeptides. Nonetheless, such polymer models still retain great predictive power, particularly for proteins in near ideal solvent conditions. As such, polymer models have shown great utility in describing the ensemble properties of chemically denatured proteins and coil-like IDPs. 

Due to the intrinsic flexibility of IDPs, models from polymer physics have proven useful to describe their ensemble properties in many specific cases. A common extrapolation from polymer models is the assumption that the conformational landscape is generally featureless and that the entire landscape is accessible. For proteins selected through evolution to fold, neither of these assumptions is true. The extent to which these assumptions hold true for IDPs depends on their class. Simple swollen coils often follow polymer scaling, but now it is recognized that many IDPs, even polar polypeptides like Huntingtin [18], undergo collapse to form more compact, disordered globules [19,20]. At these higher packing densities, self-interaction becomes more prevalent and conformational dynamics become more complex. In addition, while specific long-range intramolecular interactions are generally absent in IDPs, molecular dynamics simulations have shown that charge patterning does govern IDP conformation through intramolecular electrostatic effects [19,21,22]. Thus, sequence evolution can select for IDPs with limited or biased conformational ensembles such that a continuum of conformations is not possible.

**Figure 1:**
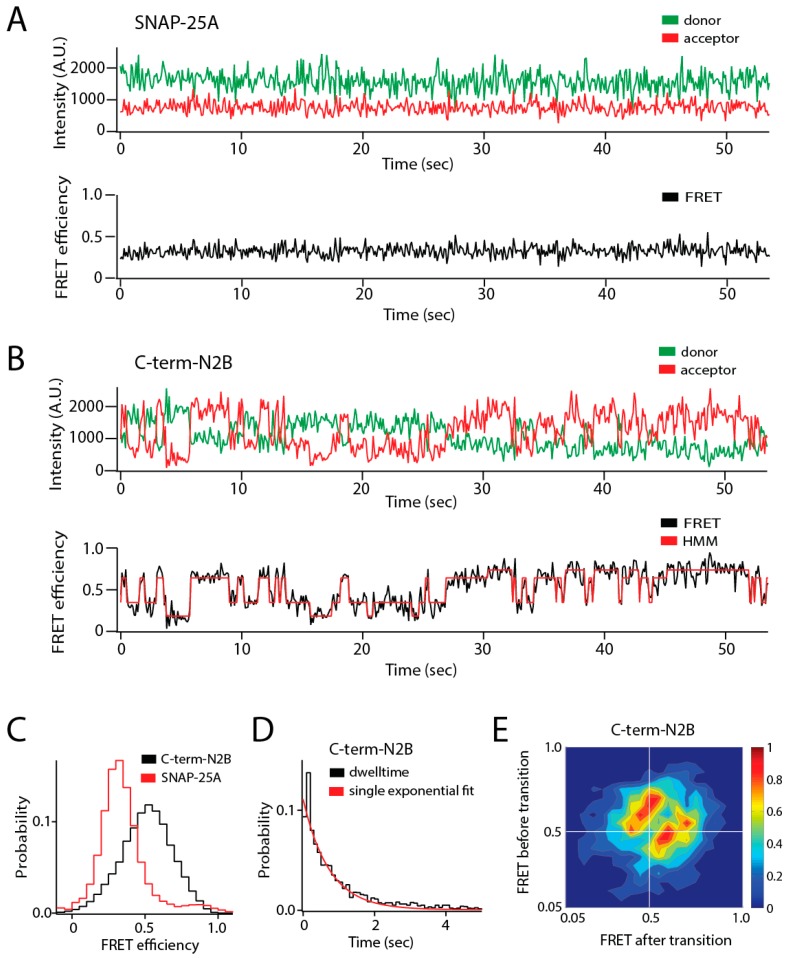
Single molecule observation of state switching in an intrinsically disordered protein (IDP) with single molecule Förster resonance energy transfer (smFRET). The soluble N-ethylmaleimide-sensitive factor activating protein receptor (SNARE) protein synaptosomal nerve-associated protein 25A (SNAP-25A) and the C-terminal tail of the GluN2B subunit of the *N*-methyl-d-aspartate (NMDA) receptor (NMDAR) (C-term-N2B) are intrinsically disordered in their native states. Residues 20 and 197 of SNAP-25A and residues 1273 and 1394 of C-term-N2B were randomly labeled with donor and acceptor fluorophores for smFRET measurements [23]. Labeled single molecules were encapsulated in liposomes (100 nm in diameter) that were then surface-tethered through biotin–streptavidin linkage on a surface passivated with biotinylated bovine serum albumin (BSA). Fluorescence emission was recorded using an emCCD (electron multiplying charge coupled device) camera at a frame rate of 10 Hz. At this time resolution, rapid conformational dynamics are time-averaged, but surface attachment allows extended observations of the same molecule for seconds to minutes. (**A**) Representative single molecule fluorescence intensity time trace of SNAP-25A (top panel) does not show spontaneous switching. The fluorescence intensities were converted to Förster resonance energy transfer (FRET) efficiency (bottom panel, black line). SNAP-25A molecules showed a stable FRET efficiency with no switching between different FRET values. (**B**) Representative single molecule fluorescence intensity time trace of C-term-N2B (top panel) and FRET efficiency (bottom panel, black line) fit by hidden Markov modeling (HMM) to obtain the dwell times in each state (bottom panel, red line). Donor and acceptor signals for C-term-N2B molecules show step-wise, anticorrelated changes in intensity, yielding steady FRET for seconds before spontaneously switching to different FRET values, which would correspond to distinct disordered states with a different average size. (**C**) FRET efficiency histogram for C-term-N2B molecules show a broad distribution across the entire range of FRET values (black line) in contrast to SNAP-25A molecules (red lines). (**D**) Histogram of state dwell times for C-term-N2B obtained from HMM. The mean state dwell time is on the order of seconds and showed no correlation with FRET efficiency. (**E**) Transition density plot for C-term-N2B shows the FRET efficiency before a transition (*y*-axis) plotted against the FRET efficiency after that transition (*x*-axis) for all observed transitions. These transitions proved too variable to resolve or assign to specific conformations. A.U.: Arbitrary units.

Conformational switching is well accepted in folded proteins, and IDPs may also be able to switch between discrete conformational ensembles while remaining disordered in both states. Here, we will focus on ensemble switching behavior identified in an increasing number of IDPs (Figure 1). In these cases, IDPs were observed to stochastically switch between distinct states within the entirety of conformational space or showed evidence of dynamics on slow timescales (Figure 1B). Both phenomena are suggestive of large energy barriers between states or the existence of metastable intermediates. A combination of methods from ensemble to single molecule is required to elucidate this individual molecular behavior. Regulating the access to distinct regions of IDP conformational space provides mechanisms to govern IDP activity. Understanding the origins of switch-like transitions will provide new insights into the mechanisms by which IDP conformational dynamics can modulate cell signaling.

## 2. Evidence of Configuration Switching in IDP Studies 

One of the best examples of state switching in polypeptides is protein folding. There are two states: the unfolded state, which is characterized by a broad ensemble of disparate, interconverting conformations, and the folded state, which is characterized by local fluctuation within a narrow range of conformational space. In most cases, such folding transitions are unidirectional under physiological conditions. However, some proteins, such as the ankyrin repeat (AR) domain from the IκB transcription inhibitor, undergo reversible order to disorder transitions at room temperature [24]. Only single molecule fluorescence resonance energy transfer (smFRET) of surface attached molecules could detect the “nanospring dynamics” that occurred on the seconds timescale as individual akyrin repeats came “unglued” [25]. Ensemble nuclear magnetic resonance (NMR) measurements on the same protein showed well-resolved NMR cross-peaks and high-order parameters but no signs of dynamic behavior [24]. Such a slow timescale for state switching suggests a large energy barrier separating these regions of conformational space.

A similar conformational state switching has been reported in α-synuclein, an IDP linked to Parkinson’s disease that undergoes a disorder-to-order transition upon interaction with amphiphilic small molecules or membranes. However, in this case, the transition is not spontaneous but regulated by functional interactions [26,27,28]. Other IDPs are known to change their form of disorder in response to physiological signals such as ion influx or posttranslational modifications like phosphorylation [29,30]. A key aspect of state switching in these IDPs is that the entire conformational landscape is not always accessible or there are sharp energy barriers, which separate discrete subregions of conformational space. The conformation is not “continuously tunable” [26].

Given the broad ensemble of conformations an IDP may adopt, it is not always possible to know how much of the conformational landscape is being explored and when such switching is occurring. One hallmark of deeper energy basins within the conformational landscape is the presence of slow timescale dynamics. However, most structural methods are not well suited for detecting slow timescale conformational dynamics.

A comparison of synaptic IDPs and IDRs revealed stochastic conformational switching in two out of five proteins in a test set despite the fact that all proteins appeared similarly intrinsically disordered by other measures [23]. The cytoplasmic domains from both neuroligin and the GluN2B subunit of the *N*-methyl-d-aspartate (NMDA) receptor (NMDAR) showed continuous hop-like conformational diffusion with Förster resonance energy transfer (FRET) shifts equivalent to nanometer scale motions in a single 100 millisecond time bin (Figure 1B). The sensitivity to stoichiometry provided by single molecule detection allows IDP clustering or aggregation to be distinguished from single molecule conformational switching. These IDRs adopted a compact globular form of disorder, yet another IDP (synaptobrevin) with a similar form of disorder failed to show transitions [23].

The transitions in GluN2B were detected with seven different FRET labeling combinations representing dye separations from 83 to 172 residues [31]. Only the shortest separation of 15 residues failed to show any transitions, which is expected because a short polypeptide segment should not be capable of large conformational changes. This important control confirms that photophysical effects on dye environment and orientation are not the origin of the transitioning phenomena. Because of their complexity, the transitions observed in synaptic IDRs proved uninterpretable in terms of structural intermediates [23].

Similar stochastic transitions between FRET efficiency levels were observed in the smFRET traces for protein 4.1, a cytoskeletal adaptor protein that stabilizes spectrin–actin crosslinks [32]. The protein appeared to switch between an unresolved number of discrete conformational states. Interestingly, while binding of protein 4.1 to the nuclear mitotic apparatus (NuMA) protein changed the pattern of transitions, it did not eliminate the transitions, indicating that the complex retains switch-like dynamics. Similarly, binding partner interactions with the synaptic scaffold protein postsynaptic density protein 95 (PSD-95) also showed no effect on conformational switching in the IDR from neuroligin [23]. Conformational switching may play a functional role even after these IDPs interact with downstream factors.

State switching in IDPs is also possible on faster timescales. The yeast prion protein Sup35 is a translation termination factor that forms amyloid fibrils. At low concentrations, smFRET showed that the protein formed a compact disordered globule [33]. However, fluorescence correlation spectroscopy (FCS) analysis of fluorescence quenching revealed at least two well separated components to the dynamics, including a slower component that originated from long-range contacts.

## 3. Theoretical Framework for Understanding IDP Conformational Switching

Swollen random coils can fully sample a “flat” energy landscape. Fast sampling is possible because little energy is needed to overcome small barriers between different configurations. Such IDPs have been described using equilibrium statistical mechanics as freely joint Gaussian chains, self-avoiding coils, and worm-like chains [17,23,34]. The conformational switching, described above in Section 2, challenges such approaches because these IDPs are sequestered off from the entirety of the conformational space. Thus, in the absence of the simplifying assumption that all states are present and equally probable, one cannot construct a statistical ensemble that represents all the possible states.

Intrinsically disordered protein conformational switching has been likened to the Lorenz attractor in nonlinear chaotic systems [35]. Like the Lorenz attractor, this switching behavior in IDPs has been shown to be sensitive to initial conditions and is also restricted to be near the attractor points in conformational space. Chaos, in dynamical mechanical systems, is dependent on underlying nonlinear relationships in the governing interactions. Intrinsically disordered proteins certainly have interactions within their peptide chain, with other components in solution, and with the host fluid that are likely to involve nonlinearities.

All conformational transitions are noise-assisted reactions of the type described by Kramers’ transition state theory [36,37,38]. The energy for excursions between basins must come from fluctuations where the polypeptide gains enough energy from the thermal milieu. Therefore, the continuum of timescales relates to the continuum of barriers between different regions of conformational space. Polypeptides are not uniform chemical polymers. Within the core of a compact, globular IDP there exists a nonlinear potential as alternate long-range interactions (favorable and unfavorable) are transiently sampled as distinct regions of the polypeptide chain are brought into close proximity. Given such a fluctuating force, thermally activated transitions could occur across a range of timescales. Even rare transitions are possible within some finite time.

Within Kramers’ theory, the time to escape a basin is related to the damping factor associated with internal friction. Studies of IDPs have pointed to the important role of internal friction in modulating conformational dynamics and folding [39,40]. Friction within expanded IDPs depends on the quality of solvent interactions. However, in collapsed, globular IDPs, shielding of residues in the interior removes solvent interactions and can create a complex network of coupled and decoupled chain segments [34,41]. Thus, nonlinear dynamics arising from feedback among these different couplings should not be surprising. Large transitions (e.g., folding–unfolding) involve a complex set of possible pathways within the energy landscape [42].

**Figure 2:**
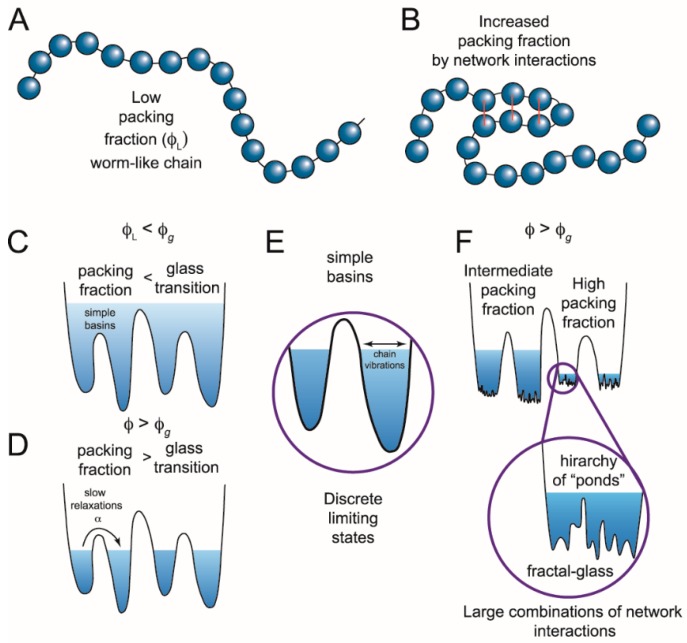
Switching in IDPs shows similarity to structural glasses. (**A**) IDPs can be modeled as worm-like chains when the packing fraction (ϕ) of the constituent particles is low (ϕ_L_). Glasses can flow when the packing fraction is low but undergo a phase transition at high packing fractions when particles are caged by interactions with their neighbors. Applying this analogy of glasses to describe IDPs, the amino acids are the constituent particles. (**B**) If amino acids interact, the packing fraction increases, giving rise to a more complex energy landscape with states at different energy levels. (**C**) These states (simple basins) represent the minima of the energy landscape. When the packing fraction is lower than the glass transition (ϕ_g_), or collapsed state, the polypeptide can sample a continuum of states following polymer models. Transitions between states would be described by Kramers’ transition state theory. (**D**) When there are many network interactions, the packing fraction increases and not all conformations are accessible. The limiting states are separated by deep wells and discrete states can be identified. Such conformational switching could result from a number of different mechanisms including internal friction, long rage potentials, ion mediated interactions, or other forces that modulate the network interactions. (**E**) Discrete limiting states produce conformational switching, yet there is still a subensemble of conformations inside each basin. (**F**) At high packing fractions, the subensemble of states can also become discontinuous, generating potential transitions across widely separated temporal domains. (Figure adapted from reference [47]).

Conformational switching occurs over a wide range of timescales, ranging from very slow processes lasting for seconds down to microsecond timescales [43]. To explain this, a scale-free approach is needed. The energy landscape we have postulated for these IDPs, containing multiple minima with high energy barriers, is also characteristic of glasses (Figure 2). Mean-field theories of disordered glasses can describe the existence of stable and metastable states [44,45,46]. At low density or low packing fraction (ϕ), glasses remain liquid and can sample all states (Figure 2C). As the packing fraction increases, the individual particles are subject to jamming and undergo a glass transition where they become trapped within individual basins (Figure 2D). In this jammed state, excitations can extend over a wide range of timescales [47].

Deep within the glass phase, each individual basin becomes a metabasin composed of a fractal hierarchy of sub-basins (Figure 2F) [48]. This fractal free-energy landscape was recently proposed to explain the roughness transition in structural glasses [47]. A similar fractal hierarchy or scale free- energy landscape could arise from packing of chain segments within an IDP. The packing particles are collapsed chain segments within the polymer along with coordinated ions and bound solvent (Figure 2B). A high packing fraction could lead to jamming and produce metastable configurations, which might switch to other equally stable configurations as intrachain interaction forces evolve with the conformational search. In this model, such local phase transitions would extend to other packed chain segments, leading to the observed IDP conformational switching across a wide temporal regime.

## 4. Possible Mechanisms to Sequester Regions of Conformational Space in IDPs

The origin of continuous slow timescale dynamics or state switching is not clear. Proline isomerization is one of the conformational changes in polypeptides known to operate on this timescale and can result in state switching. Conformational exchange rates linked to proline isomerization were detected in the IDR of the transcriptional regulator E2 from human papillomavirus (HPV) by collecting NMR nuclear overhauser spectroscopy (NOESY) spectra at different mixing times [20]. This discrete conformational transition in E2 is the rate-limiting step for antibody recognition of this viral antigen. Interestingly, proline depletion of GluN2B reduced the number of molecules that showed any stochastic FRET transitions, but proline-depleted constructs showed similar transition rates to the wild type [49]. Thus, for GluN2B, proline appeared to facilitate switching but not govern the timescale.

Aside from proline isomerization, the major determinant of IDP conformation are self-interactions (i.e., between amino acids in the chain) and solvent interactions. Most intrachain interactions in IDPs are local, yet chain segments can partition into both packed and extended forms of disorder. These chain segments are continuously exposed to one another during the conformational search, with favorable interactions restricting chain motions and unfavorable interactions limiting access to some conformations. Within the disordered globule, the quality and quantity of the solvent is evolving as water and ions interact with local chain segments and are drawn into the “core” of the IDP. For example, ions from solution could neutralize local charge densities stabilizing distinct configuration space from those visited in the absence of the ions. Compacted chain segments of IDPs can temporarily exclude water [34,41,50], which would result in stable local minima that would limit the conformational search. As noted above, the complexity of conformational space within these fractal basins would mean that available thermal energy could be dissipated by small, local rearrangements rather than long-range motions.

Chain reconfiguration could also transiently trap unfavorable interactions within the globular interior. This unfavorable interaction would destabilize any metastable local conformations and drive the system to a new region of conformational space. Reintroduction of water to a temporarily dehydrated chain segment would upset the balance of the chain interactions. Chain reconfiguration will inevitably bring charged amino acids within the globular interior with a finite probability of unfavorable interactions being trapped by approach jamming. These sorts of trapped interactions represent high energy metastable intermediates that could suddenly be released when a random fluctuation exposes a pathway that permits access to alternate regions of conformational space. Such an event would manifest as sudden stochastic changes in the sampled conformational space, or what we have termed IDP conformational switching. 

It remains to be established that effects as subtle as sequestering an additional ion or water molecule could generate the spontaneous IDP ensemble switching that is the focus of this discussion. There are some relevant examples where such small perturbations can affect molecular properties. Deoxyribonucleic acid (DNA) certainly can transiently recruit or bind ions from solution changing its polymeric properties [51], bearing in mind that DNA can easily be modeled as a worm-like chain [52,53]. Certainly, changes in proteins at the level of a single amino acid can impact disordered states [54,55,56,57,58,59,60,61,62,63]. Changes of the amino acid sequence in IDPs and IDRs have been linked to human diseases. Aggregation of α-synuclein has been linked to sequence composition in familial forms of Parkinson’s disease. Familial mutations that enhanced aggregation slowed conformational dynamics, while mutations that sped up intramolecular diffusion inhibited aggregation [64]. De novo missense mutations in the cytoplasmic IDR of the NMDAR, discussed above, are linked familial forms of epilepsy [65,66]. That changes at the level of individual amino acids can reshape the energy landscape of IDPs suggests that transient interactions with ions or water could generate the observed ensemble switching phenomenon. 

## 5. State Switching Can Occur Close to the Boundary of a Folding Transition

Most protein folding studies to date have focused on single domain proteins displaying simple two state behavior. The folding of large multidomain proteins can be more complex with metastable intermediate states in their energy landscapes. At low denaturant concentrations (0.65 M GdmCl), the three-domain, 214 amino acid protein adenylate kinase (AK) began to show stochastic FRET transitions between six states [42]. The transitions were too variable to resolve nor could they all be assigned to specific denatured intermediates. Furthermore, the “situation [became] even more complex at higher concentrations of denaturant.” [42]. This shows that a folded protein slightly destabilized by denaturants shows similarly complex transitioning to that observed in switching IDPs. 

Thus, transitioning molecules arise when the impetus for a polypeptide to fold is lowered slightly and becomes more complex as more of the conformational landscape becomes available. Ultimately, transitions become faster until they are unresolved and become continuous dynamics of the type described by polymer models. Thus, a similar transition should be possible if an expanded coil is brought close to a folded state. Interestingly, smFRET measured in the presynaptic fusion protein SNAP-25 did show scaling that fit to polymer models [23]. However, stochastic FRET switching was induced in SNAP-25 upon binding the SNARE protein syntaxin, which is a binary intermediate in the formation of the tripartite SNARE complex [67]. This confirms that an expanded coil-like IDP can be converted to state switching as protein interactions restrict access to portions of the conformational landscape.

## 6. Functional Relevance of IDP Ensemble Switching through Phosphorylation

Aside from transitions involving an ordered state, few biological functions have been directly connected to the modulation of IDP conformational ensembles. Post-translational modifications can change the interaction potential of existing amino acids [29,30,68,69,70,71]. Phosphorylation is among the best-documented mechanism for dynamically affecting biological function of proteins. In particular, phosphorylation has been connected with modulating disorder in IDPs [29,68,69,70,71,72,73,74,75].

For example, the ribonucleic acid (RNA) binding protein fused in sarcoma (FUS) forms aggregated protein deposits in neurodegenerative disorders, which are modulated by phosphorylation. Nuclear magnetic resonance studies found that phosphorylation of FUS, or phosphomimetic mutations, did not “alter the disordered structure of FUS” [50]. However, phosphorylation of FUS did decrease transient polypeptide collapse and increase the radius of gyration. These changes in the IDP ensemble were associated with reduced intermolecular interaction and aggregation such that a phosphomimetic variant reduced the toxicity of FUS to live cells [50].

**Figure 3:**
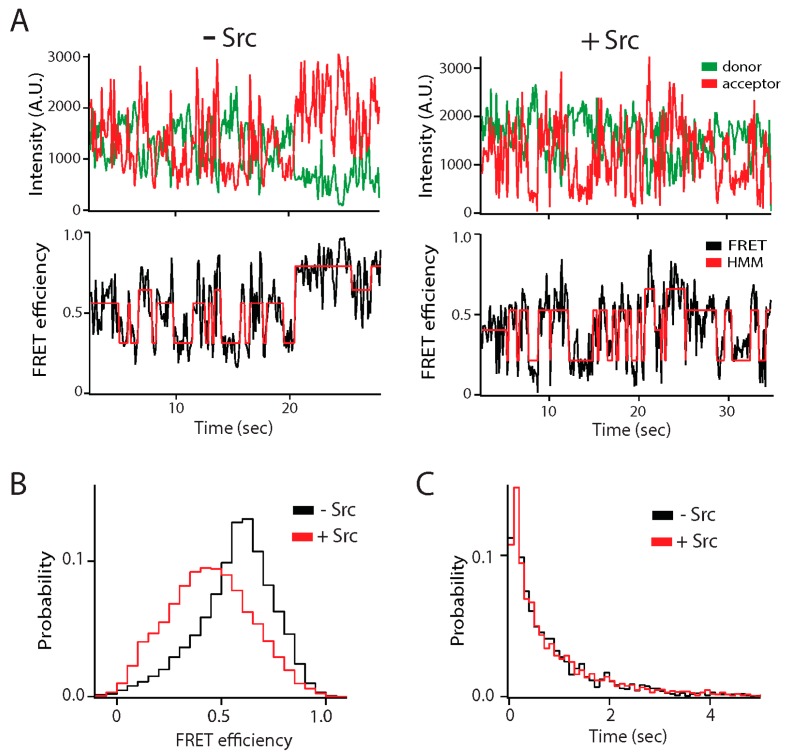
Effect of Src kinase phosphorylation on the conformational ensemble of the NMDA receptor. The C-terminal intrinsically disordered region (IDR) of the GluN2B subunit of the NMDA receptor (C-term-N2B) was randomly labeled with donor and acceptor fluorophores at residues 1323 and 1453 [31]. Src kinase phosphorylates C-term-N2B on tyrosine residues 1336 and 1472. (**A**) Representative single molecule fluorescence intensity (top panels) and fluorescence resonance energy transfer (FRET) efficiency fit by hidden Markov modeling (HMM) (bottom panels) for unphosphorylated (left) and phosphorylated (right) C-term-N2B. Phosphorylation did not induce structure in C-term-N2B as the dynamic transitions continued. (**B**) FRET efficiency histogram of C-term-N2B before (black line) and after (red line) Src phosphorylation. Phosphorylation led to shift towards lower FRET indicating a general expansion of the polypeptide, which was confirmed with hydrodynamic measurements. (**C**) Dwell time histograms for transitions in C-term-N2B, obtained from HMM, before (black line) and after (red line) Src phosphorylation. Although phosphorylation shifted the ensemble FRET efficiency, stochastic transitions were unaffected. A.U.: Arbitrary units.

Phosphorylation of an IDR was also linked to biological function of the NMDAR, which uses the energy of neurotransmitter binding to open its ion channel. Allosteric inhibition of channel gating by extracellular zinc can be alleviated by Src kinase phosphorylation of the C-terminal IDR of the GluN2B subunit (C-term-N2B) [76], which switches conformation as noted above (Figure 3A) [23]. The effect of Src phosphorylation is mediated by expansion of this globular IDR without affecting conformational transitions (Figure 3B,C) [31]. Deleting prolines near the phosphorylation sites had the opposite effect and compacted the IDR while reducing the probability of transitions. Both of these modifications, which change the size of the disordered states in opposite directions, eliminated the ability of zinc to allosterically regulate channel gating without disrupting the underlying gating mechanisms [49]. Thus, this IDR appears to have an optimal packing density that supports allosteric coupling between domains of the receptor.

Phosphorylation-induced modulation of an IDP ensemble was also connected to regulation of cellular signaling processes in prostate cancer. Prostate-associated gene 4 (PAGE4) is an IDP that is expressed exclusively in adult males who have prostate cancer. Interactions between PAGE4 and transcription factors have been suggested to control androgen sensitivity [77,78,79,80,81,82,83]. Both homeodomain-interacting protein kinase 1 (HIPK1) and CDC-Like Kinase 2 (CLK2) phosphorylate PAGE4, with CLK2 modifying many more sites. Experiments combining NMR, paramagnetic relaxation enhancement (PRE), small angle X-ray scattering (SAXS) and smFRET have determined that HIPK1 phosphorylation compacts the ensemble, while CLK2 phosphorylation leads to expansion. Molecular dynamics (MD) simulations linked changes in PAGE4 ensembles to distinct phosphorylation patterns [84]. These different phosphorylation patterns influenced transcription factor binding. HIPK1-treated PAGE4 binds to AP-1, whereas CLK2 treatment of PAGE4 decreases its affinity for AP-1. Phosphorylation by HIPK1 effectively disrupts the PAGE4 interaction with c-Jun and consequently stimulated c-Jun dependent transcription in prostate cancer cell models [85,86]. In contrast, CLK2 phosphorylation of PAGE4 inhibited c-Jun dependent transcription. Importantly, HIPK1 is expressed in both androgen-dependent and androgen-independent prostate cancer cells, whereas CLK2 and PAGE4 are expressed only in androgen-dependent cells. A model for the PAGE4–Jun-Fos (AP-1)–AR regulatory circuit suggests phosphorylation patterns in prostate cancer cells can oscillate. Thus, it was proposed that androgen dependence may vary in time [78] with switching between androgen-dependent and androgen-independent phenotypes being a result of details of PAGE4 phosphorylation [77,79,80,84]. This differential phosphorylation is associated with opposing shifts in the conformational ensemble of PAGE4.

## 7. Conclusions and Prospects for Understanding IDP Switching

Intrinsically disordered proteins are essential components of cellular signaling pathways because of their adaptability to the local environment. Cell signaling events can lead to changes in ionic composition or pH that affect solvent quality while posttranslational modifications affect net charge and hydropathy. Intrinsically disordered proteins can respond instantly to such signals by shifting to alternate conformational ensembles. Their unique capabilities can allow a single IDP to interact with multiple binding partners. Intrinsically disordered proteins also play structural roles as linkers or entropic elements. Additionally, IDPs are linked to the formation of membraneless organelles (i.e., liquid phase separation) [87,88]. These important functions allow for more nuanced coordination of signal transduction. 

Here, we have highlighted a recently identified conformational switching phenomenon observed in a small but growing number of IDPs. Intrinsically disordered proteins in this class appear disordered by standard measures of secondary structure or hydrodynamic mobility but also appear to fluctuate between well-separated regions of conformational space. If the conformational ensembles are functionally distinct, then mechanisms that biases conformational sampling will govern protein activity. We suggest such control over IDP switching may be an important regulator of cellular signaling networks. 

Few experimental methods are sensitive to conformational switching in IDPs [89]. Integrative structural biology approaches where several methods are applied to a single protein may help inform our understanding of the molecular mechanisms controlling intrinsic disorder in proteins. Several methods including smFRET, NMR, electron paramagnetic resonance double electron–electron resonance (EPR-DEER), paramagnetic relaxation enhancement (PRE), and small-angle neutron scattering (SANS)/small-angle X-ray scattering (SAXS) have all been applied to IDP studies. These different methods are able to provide complementary information about local chain contacts and global disordered structure. Comparing and cross-validating such results with multiple methods may help reveal specific interactions that are critical for determining details of the disordered state. To date, switching has only been observed in vitro, so it remains to be determined if such switching transitions are present within live cells [3], where smFRET may enable direct observation [90,91,92]. Similarly, switching has been primarily studied under dilute conditions so it remains unknown whether the phenomenon persists in the condensed phase [93,94].

In addition to experimental approaches, MD simulation is a critical tool to understand the mechanisms that govern access to the full conformational ensemble. Molecular dynamics simulations of IDPs are particularly challenging. Details of the force fields are critically important and are a topic of continued development [95,96]. The long timescales required to observe ensemble switching are difficult to achieve for standard MD simulation and require more sophisticated ensemble sampling approaches. Despite these difficulties progress applying MD simulation to IDPs is an area of active work [84,97,98,99,100,101,102,103]. Critically, experimental studies and simulations provide essential feedback to each other [103,104,105,106,107]. The molecular mechanisms controlling rapid dynamics in IDPs are becoming clearer through MD simulation [83,101]. However, spontaneous ensemble switching of an IDP has yet to be reported so molecular details remain unknown.

If IDP ensemble switching does regulate signaling, then these mechanisms could be used for therapeutic intervention in those pathways. Efforts are already underway to identify small molecules that can specifically bind IDPs [2,108,109]. An exciting functional connection has been found for the transcription factor TFIID where a drug-like molecule affected the DNA interaction to prevent transcription initiation by RNA polymerase [2,109]. Additional strategies for intervening in signaling pathways within the diseased state will likely emerge as our understanding grows as to how IDP conformational dynamic leads to function within cellular networks.
